# Differential neuro-immune patterns in two clinically relevant murine models of multiple sclerosis

**DOI:** 10.1186/s12974-019-1501-9

**Published:** 2019-05-22

**Authors:** Krista D. DiSano, Michael R. Linzey, Darlene B. Royce, Andrew R. Pachner, Francesca Gilli

**Affiliations:** 1 Department of Neurology, Dartmouth Hitchcock Medical Center and Geisel School of Medicine, One Medical Center Drive, Lebanon, NH 03756 USA; 2 Program in Experimental and Molecular Medicine, Dartmouth College, Hanover, NH USA

**Keywords:** Multiple sclerosis, Immunoglobulins, B cells, EAE, TMEV-IDD

## Abstract

**Background:**

The mechanisms driving multiple sclerosis (MS), the most common cause of non-traumatic disability in young adults, remain unknown despite extensive research. Especially puzzling are the underlying molecular processes behind the two major disease patterns of MS: relapsing-remitting and progressive. The relapsing-remitting course is exemplified by acute inflammatory attacks, whereas progressive MS is characterized by neurodegeneration on a background of mild-moderate inflammation. The molecular and cellular features differentiating the two patterns are still unclear, and the role of inflammation during progressive disease is a subject of active debate.

**Methods:**

We performed a comprehensive analysis of the intrathecal inflammation in two clinically distinct mouse models of MS: the PLP_139-151_-induced relapsing experimental autoimmune encephalomyelitis (R-EAE) and the chronic progressive, Theiler’s murine encephalomyelitis virus-induced demyelinating disease (TMEV-IDD). Microarray technology was first used to examine global gene expression changes in the spinal cord.

Inflammation in the spinal cord was further assessed by immunohistochemical image analysis and flow cytometry. Levels of serum and cerebrospinal fluid (CSF) immunoglobulin (Ig) isotypes and chemokines were quantitated using Luminex Multiplex technology, whereas a capture ELISA was used to measure serum and CSF albumin levels. Finally, an intrathecal Ig synthesis index was established with the ratio of CSF and serum test results corrected as a ratio of their albumin concentrations.

**Results:**

Microarray analysis identified an enrichment of B cell- and Ig-related genes upregulated in TMEV-IDD mice. We also demonstrated an increased level of intrathecal Ig synthesis as well as a marked infiltration of late differentiated B cells, including antibody secreting cells (ASC), in the spinal cord of TMEV-IDD, but not R-EAE mice. An intact blood-brain barrier in TMEV-IDD mice along with higher CSF levels of CXCL13, CXCL12, and CCL19 provides evidence for an intrathecal synthesis of chemokines mediating B cell localization to the central nervous system (CNS).

**Conclusions:**

Overall, these findings, showing increased concentrations of intrathecally produced Igs, substantial infiltration of ASC, and the presence of B cell supporting chemokines in the CNS of TMEV-IDD mice, but not R-EAE mice, suggest a potentially important role for Igs and ASC in the chronic progressive phase of demyelinating diseases.

**Electronic supplementary material:**

The online version of this article (10.1186/s12974-019-1501-9) contains supplementary material, which is available to authorized users.

## Introduction

Multiple sclerosis (MS) is a chronic inflammatory disease of the central nervous system (CNS), which causes demyelination, axonal loss, and progressive disability. The cause of MS is unknown, but one hypothesis is that overactivity of both the innate and adaptive arms of the immune system may be involved in the activation of self-reactive or cross-reactive immune cells to antigens associated with the myelin sheath and oligodendrocytes [1–4]. CD4^+^ T helper 1 (Th1) and Th17 cells, in particular, have been implicated in MS disease pathogenesis, but a variety of other cell types such as CD8^+^ T cells, B cells, monocytes, neutrophils, macrophages, and microglia have been suggested to be involved both outside of and within the CNS [5–10]. Notably, recent success with B cell depletion therapies has revitalized efforts to understand the pathogenic role of B cells in MS [11, 12].

There are two main disease patterns of MS: relapsing-remitting and progressive [13]. The relapsing-remitting disease course is characterized by clearly defined clinical exacerbations associated with the development of focal inflammatory lesions in the CNS. In contrast, in the chronic progressive course, there is increasing neurologic dysfunction thought to reflect ongoing neurodegenerative processes. To date, the molecular and cellular features differentiating the two patterns are unclear, and the role of inflammation during progressive disease is a subject of active debate.

Mouse models of human MS are helpful in identifying potential pathogenic mechanisms of the disease and its different stages. These MS models were established according to the various hypotheses of the pathogenesis of MS and are aimed at reproducing the clinical signs of MS as far as possible. The two most commonly studied animal models of MS are (1) the autoimmune-induced experimental allergic/autoimmune encephalomyelitis (EAE) [14] and (2) the virally-induced chronic Theiler’s murine encephalomyelitis virus-induced demyelinating disease (TMEV-IDD) [15, 16].

EAE is a well established and widely used model of MS, which involves using defined myelin antigens to induce an encephalitogenic immune response. Different manifestations characterize the disease, depending on the antigen used to induce EAE and the rodent strain. In the SJL mouse strain, for example, EAE is usually induced by immunization with a myelin proteolipid protein peptide (PLP_139–151_) or the adoptive transfer of PLP-specific CD4^+^ T cells into syngeneic recipients [16, 17]. PLP-induced EAE most strongly resembles the relapsing-remitting form of MS (R-EAE), thus representing a valuable model to study the development of relapses and acute inflammation.

TMEV-IDD is another inflammatory model of MS. Different from R-EAE, TMEV-IDD results from a chronic virus infection in the CNS, associated with significant intrathecal antibody production [18–20]. Overall, the TMEV-IDD model features a chronic progressive disease course that lasts for the entire lifespan in susceptible SJL mice [21]. Several features of progressive MS, including the role and significance of axonal injury and repair [22, 23], the partial independence of disability from demyelination [24], and the importance of remyelination [25], have all been demonstrated in this model. The validity of a viral model of MS is also supported by epidemiological evidence that suggests the possibility that a virus may play a role in or even be an etiologic factor for the development of MS [26, 27]. Finally, TMEV-IDD has been widely used to demonstrate mechanisms by which viral infection may contribute to the generation of a heightened immune response, leading to chronic inflammation in the CNS and neurodegeneration.

Although both R-EAE and TMEV-IDD have significantly advanced our understanding of MS and have shed light on some mechanisms underlying relapses and progression in MS, there is an almost total lack of comparative studies between the two animal models. Exploring both similarities and differences between R-EAE and TMEV-IDD, i.e., a relapsing-remitting and a progressive model of MS, may lead to a better understanding of the pathological processes leading to a relapsing-remitting clinical course rather than a progressive disease course.

## Materials and methods

### Animal models of MS

The experiments consisted of the analysis of four different experimental groups of SJL/J mice: TMEV-IDD, i.e., TMEV-infected SJL/J mice; R-EAE, i.e., PLP-immunized SJL/J mice; sham-treated TMEV-IDD age-matched SJL/J controls; and sham-treated R-EAE age-matched SJL/J controls.

SJL mice were all purchased from The Jackson Laboratory (Bar Harbor, ME) to reduce variability. Mice were maintained on standard laboratory chow and water *ad libitum*, and standard clinical assessments like weight loss and clinical grading of disease were regularly performed over the follow-up.

### Induction of TMEV-IDD

TMEV-IDD mimics some of the pathological and clinical features observed in patients with a progressive form of the disease [21]. TMEV-IDD was induced by injecting 2 million plaque-forming units (PFU) of TMEV, strain BeAn, into the right cerebral hemisphere of 6-week old female SJL/J mice. Mice were first anesthetized with isofluorane and inoculated by free-hand injection in a 30-μL final volume. PFU were determined by a cytopathic effect assay (CPE). Blood from each experimental and control mouse at days 30 and 90 post-infection (p.i) was collected from the retro-orbital plexus of anesthetized mice; serum was isolated and stored at − 80 °C. Neurological disability was assessed weekly using the Rotamex Rotarod instrument (Columbus Instruments; Columbus, OH) as previously described (Additional file 1: Table S1) [28]. Mice were necropsied around day 120 p.i, or earlier if their severe neurological disability endangered their life (Additional file 1: Table S1). Necropsy techniques, including anesthesia, intracranial injection, perfusion with phosphate-buffered saline (PBS), and the collection of blood, cerebrospinal fluid (CSF), and other tissues such as spinal cord and brain, were performed as previously described [19, 29].

Virus persistence in the CNS is necessary to develop the chronic progressive demyelinating disease, which is consistently induced in 90% of the infected mice [16]. Therefore, in our analyses, we only included mice effectively infected with TMEV. Effective infection was proven by demonstrating viral infection of the spinal cord at necropsy by real-time reverse transcription quantitative PCR (RT-qPCR) for TMEV mRNA [30, 31] and anti-TMEV antibody in serum by ELISA [31, 32]. Levels of serum anti-TMEV antibody were tested at 30, 90, and 120 days p.i to further confirm viral persistence.

### Induction of myelin PLP-induced R-EAE

The PLP peptide-induced R-EAE model in SJL/J mice was chosen because it represents a well-suited tool to experimentally address mechanisms that cause the relapsing autoimmune pathology of the CNS in MS [16]. R-EAE was induced in 8-week-old SJL/J female mice by immunization with PLP_139-151_ peptide injection in complete Freund’s adjuvant (CFA) containing 1 mg/mL *Mycobacterium tuberculosis*. Mice were immunized according to a standard protocol using the Hooke Kit™ PLP_139-151_/CFA emulsion pertussis toxin (PTx) (Hooke Labs, Lawrence, MA, USA), which contains 200 μg myelin proteolipid protein (PLP_139–151_) in 200 μL CFA. The emulsion was injected subcutaneously at 4 sites on the back, followed by an intraperitoneal injection of 600 ng of PTx in 100 μL of PBS. Mice were scored blindly and daily from day 7 post-immunization. R-EAE disease was scored using a 5-point grading with 0 for no clinical disease, 1 for tail weakness, 2 for paraparesis (incomplete paralysis of one or two hindlimbs), 3 for paraplegia (complete paralysis of one or two hindlimbs), 4 for paraplegia with forelimb weakness or paralysis, and 5 for moribund or dead animals (Additional file 1: Table S1) [16]. R-EAE mice were sacrificed at the peak of disease severity (around day 15 post-immunization) or when presenting a clinical score ≥ 3 as per our IACUC protocol (Additional file 1: Table S1). Techniques used in the necropsies were performed as described above.

### Control mice

Control mice in these experiments were included carefully to reflect differences between R-EAE and TMEV-IDD, taking into account differences with respect to age at induction and age at maximal disease. According to previous studies [16], the best age for induction of R-EAE in mice is 8 to 12 weeks, whereas TMEV-IDD development is expected to be most prevalent in mice infected at 6 to 7 weeks. Typically, TMEV-infected mice begin to show signs of clinical disease between 30 and 40 days p.i. and develop progressive disability over an average of 120 days p.i., i.e., at ~ 24 weeks of age. Conversely, R-EAE mice experience their first relapse much earlier, with the most intense symptoms peaking around day 15 post-immunization, i.e., at ~ 10 weeks of age. This age bias represents a commonality between the two mouse models and human disease, as progressive forms of MS affect, on average, older people compared to relapsing MS. On the other hand, the age-related changes of the immune system may represent a significant confounding factor for biological analyses. Hence, in the present work, age-related bias was specifically addressed by adding two different control groups, i.e., 10 weeks old and 24 weeks old mice, to be compared to their respective age-matched experimental group, i.e., R-EAE and TMEV-IDD, respectively. Each pairwise comparison between the control group and its respective age-matched experimental group was analyzed to identify unique patterns independent of age-related effects. Eight weeks old and 6 weeks old SJL/J mice were sham-treated with equal volumes of PBS and followed over time in parallel to the respective experimental groups, i.e., R-EAE and TMEV-IDD, respectively (Additional file 1: Table S1).

### Total RNA extraction from spinal cord tissues

Total RNA was extracted from dissected snap-frozen lumbar and sacral portions of the spinal cords with TRIzol® reagent (Ambion, Austin, TX), according to the manufacturer’s protocol. RNA was precipitated from the aqueous phase by addition of isopropanol, washed with ethanol and solubilized for final storage at − 80 °C until further use. RNA concentration and purity were assessed by spectrophotometry (NanoDrop ND1000; NanoDrop Technologies, DE). For microarray analyses, RNA integrity was further evaluated using the Agilent 2100 Bioanalyzer and its lab-on-a-chip platform technology (Agilent Technologies UK Ltd, West Lothian, UK).

### Microarray analysis of gene expression in spinal cord tissues

Microarray analysis was performed with the Illumina MouseWGE6 BeadChip (Illumina, London, UK). All RNA samples were analyzed on the same day under identical conditions. RNA samples were prepared for array analysis using the Illumina TotalPrep-96 RNA Amplification Kit, following the manufacturer’s instructions (Ambion/Applied Biosystems, Warrington, UK). First and second strand cDNA was synthesized from 0.1 to 0.5 μg of total RNA and labeled with biotin. The biotin-labeled cRNA was applied to the arrays using the whole-genome gene expression direct hybridization assay system from Illumina. Finally, the BeadChips was scanned using the Illumina BeadArray Reader. The data was extracted using BeadStudio 3.2 (Illumina, London, UK).

### RT-qPCR analysis of gene expression in spinal cord tissues

Chitinase-3-like protein 4 (Chi3l4), chemokine (C-X-C motif) ligand 13 (CXCL13), and immunoglobulin-G1 (IgG1) mRNA quantitation were performed using RT-qPCR analysis of cDNA. Briefly, total RNA (50 ng/μL) was reverse transcribed with the qScript™ cDNA SuperMix (QuantaBio, Gaithersburg, MD). cDNA was then used as a template for the RT-qPCR analysis based on the 5′ nuclease assay with the PerfeCTa qPCR FastMix II ROX (QuantaBio, Gaithersburg, MD). Mouse glyceraldehyde phosphate dehydrogenase (GAPDH) was used as a reference gene. TaqMan Gene Expression Assays (ThermoFisher Scientific, Waltham, MA) were used as primers and probes for Chi3l4, CXCL13, and GAPDH, whereas custom primers and probes were used for the amplification of IgG1 [19]. The relative mRNA expression level of each target gene was analyzed by the 2^-ΔCt^ method, in which ΔCt is (Ct_target_-Ct_GAPDH_) [33].

### Protein measurements by ELISAs

Soluble proteins were extracted from spinal cord samples following the TRIzol® (Ambion, Austin, TX) method for protein isolation during RNA isolation. Proteins were precipitated from the organic phase with ethanol and isopropanol, washed with ethanol and solubilized, for final storage at − 80 °C until further use.

We measured the total amount of protein extracted with the Pierce BCA Protein Assay Kit (ThermoFisher Scientific, Waltham, MA). Specific proteins levels were then measured by using commercially available ELISAs for the quantification of Chi3l4 (LSBio, Seattle, WA) and CXCL13 (BioLegend, San Diego, CA). Values were normalized to the total amount of protein contained in each sample.

Albumin was measured in serum and CSF specimens with a commercial Mouse Albumin capture ELISA kit (Novus Biologicals, Littleton, CO) using the protocol suggested by the manufacturer.

### Protein measurements by Luminex

A magnetic bead-based approach was used to measure a broad panel of proteins in matched serum and CSF specimens, including the immunoglobulin isotyping panel and reagents kit (MilliPlex MAP Mouse Immunoglobulin Isotyping Magnetic Bead Panel, EMD Millipore, Burlington, MA) and the chemokine Luminex kit (Bio-Plex Pro™ Mouse Chemokine Panel, BioRad, Cambridge, MA). To differentiate blood- *vs*. CNS-derived Igs in the CSF, we used albumin quotient (Q_albumin_) and Ig index. Ig indices were calculated as Q_Ig_/Q_albumin_ = [(CSF/serum Ig)/(CSF/serum albumin)].

### Immunophenotyping of spinal cord-infiltrating B cells through flow cytometry

For B cell analysis via flow cytometry, spinal cord-derived mononuclear cells were isolated from individual spinal cords utilizing collagenase type I (1 mg/mL) and DNase I (100 U/mL) (Worthington Biochemical Corporation, Lakewood, NJ) as previously described [34]. Cells were recovered from the 30/70% Percoll interface and resuspended in fluorescence-activated cell sorter (FACS) buffer for flow cytometry staining. Before staining, cells were incubated with fixable viability stain 780 APC-Cy7 (BD Biosciences, San Jose, CA) and washed in FACS buffer. Cells were then incubated with FACS buffer supplemented with 1% mouse serum and 0.5 μl per 10^6^ cells rat anti-mouse FcγIII/II mAb (2.4G2; BD Bioscience, San Jose, CA) to prevent non-specific staining. Expression of cell surface markers was assessed by staining with antibodies specific for CD45 (30-F11; PerCP-Cy5.5), CD19 (1D3; PE-CF594), IgD (11-26; APC), and IgM (eB131-15F9; PE) (BD Biosciences, San Jose, CA). Cells were analyzed using a Beckman Coulter Gallios flow cytometer (BD Biosciences, San Jose, CA) and FlowJo version 9.7.6 software (Tree Star, Ashland, OR). Cell numbers were calculated based on live cell yields and percentages of gated live cells. Dead cells were excluded using a fixable viability stain and comprised less than 10% of isolated cells. Doublets were excluded based on FSC-Area and FSC-Height.

### Immunohistochemical detection of B cells in spinal cord tissue

Spinal cords from PBS-perfused R-EAE or TMEV-IDD mice were fixed in 10% neutral buffered formalin for 24–48 h. Spinal cords were segmented into cervical, upper thoracic, and lower thoracic regions and embedded in paraffin. Tissue blocks were then surface decalcified using nitric acid bone decalcifier (StatLab medical products, McKinney, TX) for 5–10 min, washed, and cut in sections of 4 μm thickness. We optimized a deparaffinization and rehydration protocol performing two xylene 10-min washes followed by two 10-min 100% ethanol washes, and sequential 5-min washes of 95% ethanol, 70% ethanol, and 50% ethanol. Following PBS washes, antigen retrieval was performed at 95 °C for 30 min in Tris-EDTA 0.1% Tween 20 buffer and then cooled, washed in PBS, and blocked with 5% bovine serum albumin (BSA) and 10% goat serum. After blocking, spinal cord sections were incubated with rat IgG anti-mouse B220 mAb (BD Biosciences, San Jose, CA) or goat anti-mouse IgG (subclasses 1, 2a, 2b, 3) conjugated with Alexa Fluor 594 (Jackson Laboratories, Sacramento, CA), and rabbit IgG anti-mouse CD3 SP7 clone polyclonal Ab (Abcam, Cambridge, MA). B220 and CD3 primary antibodies were detected using secondary Alexa Fluor 594 goat anti-rat and Alexa Flour 488 goat anti-rabbit antibody (Life Technologies, Grand Island, NY). Sections were mounted with Vectashield Hardset reagent with 4′,6-diamidino-2-phenylindole (DAPI) (Vector Labs, Burlingame, CA) and examined using a Zeiss LSM 800 confocal microscope with Airyscan (Zeiss, Oberkochen, Germany). Z-series images were collected every 0.2 μm covering a tissue depth of 2–3 μm. Projected images using ImageJ software (NIH, http://rsbweb.nih.gov/ij) were supplemented with the FIJI plugin set (http://fiji.sc).

### Statistical analyses

#### Microarray statistical analysis

Statistical analysis of microarray data was performed using BRB-Array Tools version 4.2.1 (NIH, https://brb.nci.nih.gov/BRB-ArrayTools). Four biological replicate samples per experimental group were analyzed. Variance stabilizing transformation and robust spline normalization was applied to raw intensity data, which was then filtered to remove non-detectable spots as determined by Illumina Software.

The goal of the statistical analysis was to identify the subset of genes that were differentially expressed in the spinal cord between sham controls vs. R-EAE or TMEV-IDD mice. To accomplish this goal, we employed both univariate and multivariate statistical and computational methods in a supervised manner. For the univariate analysis, we used the unpaired *t* test, the non-parametric Wilcoxon rank-sum test, and the statistical analysis of microarrays (SAM) test. We then constructed generalized Venn diagrams of the statistical results using the VennMaster software package and selected differentially expressed genes as those that were considered significant by two or more of the univariate statistics. Hierarchical cluster analysis of significant genes was performed using a variety of distance metrics (Pearson, Euclidean, Manhattan, and Maximum) and clustering methods (Average, Single, Complete) for characterizing the correlations structure of the genes and the samples. Heat maps were used to visualize the clustering results. We then carried out a supervised multivariate analysis of the selected genes using decision trees (DT). The advantage of using DT is that the models can be represented as simple IF-THEN rules (e.g., IF gene 1 < 10 and gene 2 > 15 THEN disease) that are easy to interpret. The predictive ability of our multivariate models was assessed using leave-one-out cross-validation (LOOCV). The LOOCV procedure provides unbiased estimates of the prediction error or testing accuracy of the models. All analyses were carried out using the open-source R statistical package (https://www.r-project.org) and the open-source Weka data mining software package (https://www.cs.waikato.ac.nz/ml/weka/).

To address the age-related bias, we analyzed two different control groups (i.e., 10-week-old and 24-week-old mice) compared to age-matched experimental groups, i.e., R-EAE and TMEV-IDD, respectively. Each pairwise comparison between the control group and the respective age-matched experimental group was then analyzed to identify unique expression patterns independent of age-related effects.

To address the problem of small sample size, i.e., *n* = 4 samples per group, we incorporated information from diverse sources to analyze the microarray data to improve the predictability of significant genes. We used a transformed dataset, including statistical parameters, literature mining, and gene ontology data. The proposed analysis method allowed the transformation of a high dimensional microarray data into a concise and significant dataset.

To gain insight into the biological significance of the different sub-clusters of genes, we evaluated enriched functional annotation categories (Gene Ontology—GO) for the target genes using the Database for Annotation, Visualization, and Integrated Discovery (DAVID) bioinformatics tools (https://david.ncifcrf.gov) according to their enrichment *p* value.

#### Other statistical analyses

Further data generated within the present study, e.g., RT-qPCR, ELISAs, and Luminex data, were analyzed using both parametric and non-parametric statistical tests, based on the specific experimental condition. Data were compared by Student’s *t* test, i.e., Mann-Whitney *U* test, Wilcoxon test, Kruskal-Wallis test, and Friedman test. Those analyses were performed using Prism version 7.00 for Mac (GraphPad, San Diego CA), and all reported *p* values were based on two-tailed statistical tests, with a significant level of 0.05.

## Results

### Differential spinal cord gene expression in R-EAE and TMEV-IDD mice

For each mouse model, gene expression in the spinal cord of treated and control animals was compared with each other (Additional file 1: Table S1). This design yielded two main comparisons: (1) R-EAE *vs*. age-matched controls and (2) TMEV-IDD *vs*. age-matched controls (Additional file 2: Table S2). We focused on the more highly altered genes, filtering for a fold change ≥ 2 and a *p* value < 0.05. This analysis reduced the TMEV-IDD list to 526 probes and the R-EAE list to 1357 probes. The following union of the two lists led to 1494 significantly different probes, i.e., at least 2-fold up- or downregulated between control and treated mice, in at least 1 of the 2 groups.

**Fig. 1:**
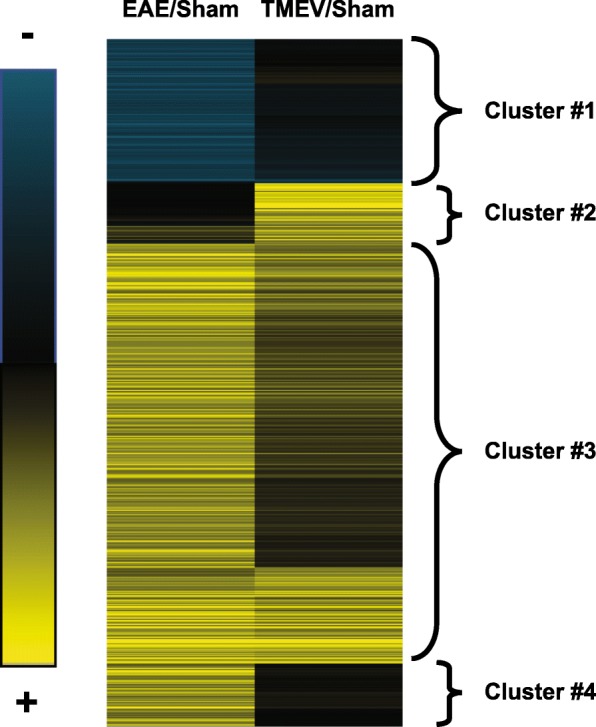
Heat map of genes differentially expressed between TMEV-IDD and R-EAE mice and their respective age-matched controls. Blue indicates an expression below the mean value, black near the mean value, and yellow above the mean value. Four different sub-clusters were identified. Sub-cluster #1 contains genes that do not change in TMEV-IDD but are downregulated in R-EAE. In sub-cluster #2, genes are upregulated in TMEV-IDD only. Sub-cluster #3 includes genes upregulated in both TMEV-IDD and R-EAE. In sub-cluster #4, genes are upregulated in R-EAE only

Next, to examine differences in altered genes between TMEV-IDD and R-EAE, we clustered the 1494 probes. We identified four sub-clusters (Fig. 1, Additional file 3: Table S3) that showed the most differential expression patterns between TMEV-IDD and R-EAE. Sub-cluster #1 contains genes that are unchanged in TMEV-IDD but are downregulated in R-EAE. In sub-cluster #2, genes are upregulated in TMEV-IDD only. Sub-cluster #3 includes genes upregulated in both TMEV-IDD and R-EAE, whereas in sub-cluster #4, genes are upregulated in R-EAE only.

In sub-cluster #1, a significant change in expression was seen in 51 known genes and 3 predicted genes, when R-EAE mice were compared with their age-matched controls. Overall, we found a total of 9 GO terms including “myelination” (*p* = 0.008), “brain development” (*p* = 0.01), and “axonogenesis” (*p* = 0.02) (Additional file 4: Table S4).

In sub-cluster #2, a significant change in expression was observed in 50 genes and 27 predicted genes, when TMEV-IDD mice were compared with their age-matched control mice. A total of 32 GO terms, including “positive regulation of B cell activation” (*p* < 0.0001), “B cell receptor signaling pathway” (*p* < 0.0001), “complement activation-classical pathway” (*p* < 0.0001), and “innate immune response” (*p* = 0.015), were significantly enriched in the set of specific TMEV-IDD upregulated genes (Additional file 4: Table S4). More specifically, many Ig-related genes such as IgK-C, Igh-VJ558, Igl-V1, and Igh-4 were upregulated in TMEV-IDD only. CXCL13, a major chemoattractant for B cells, was also overexpressed in the spinal cord of TMEV-IDD mice.

The genes upregulated in R-EAE but not in TMEV-IDD in sub-cluster #4 were 107 plus 7 predicted genes. A total of 86 GO terms, including “chemotaxis” (*p* < 0.0001), “leukocyte migration” (*p* = 0.0006), “cellular response to interleukin-1” (*p* < 0.0001), “T cell differentiation involved in immune response” (*p* = 0.019), “T helper 1 cell differentiation” (*p* = 0.024), and “adaptive immune response” (*p* = 0.030), were significantly enriched in this set of genes (Additional file 4: Table S4). Among the others, the chitinase family (Chi3l4, Chi3l3, Chi3l1) was found overexpressed in R-EAE and not in TMEV-IDD.

We also determined GO terms that were enriched in sub-cluster #3. A total of 437 GO terms, including “antigen processing and presentation” (*p* < 0.0001), “Myd88-dependent toll-like receptor signaling pathway” (*p* < 0.0001), “microglial cell activation” (*p* < 0.0001), “response to interferon-beta” (*p* < 0.0001), “Toll-like receptors signaling pathways” (TLR7, TLR3, TLR4, TLR9, all *p* ≤ 0.013), and “astrocytes development” (*p* = 0.016), were significantly enriched in the set of genes upregulated in both R-EAE and TMEV-IDD (Additional file 4: Table S4).

### Gene and protein expression confirmatory analysis

**Fig. 2:**
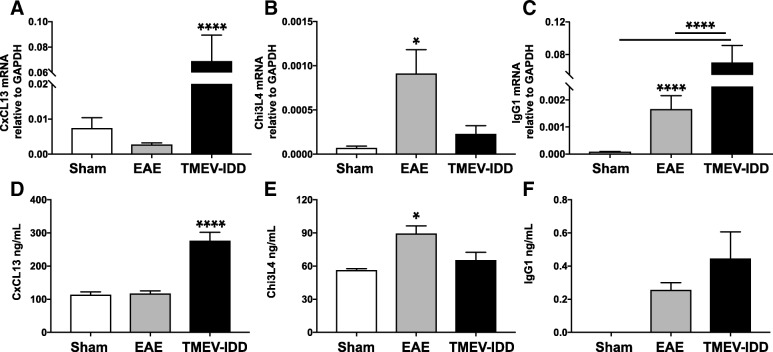
Quantitative analysis of CXCL13, Chi3l4, and IgG1 expression in spinal cord samples from TMEV-IDD and R-EAE mice. Both RT-qPCR for mRNA and ELISA for protein of CXCL13 (**a**, **d**) and IgG1 (**c**, **f**) showed significant increases in expression in TMEV-IDD mice *vs*. both R-EAE and control mice. Conversely, both RT-qPCR for mRNA (**b**) and ELISA for protein (**e**) of Chi3l4 showed increased expression in R-EAE mice *vs*. both TMEV-IDD and control mice. RT-qPCR results are representative of 3 independent experiments (*n* = 31) for TMEV-IDD and 3 independent experiments (*n* = 16) for R-EAE. Likewise, ELISA results are representative of 3 independent experiments (*n* = 15) for TMEV-IDD and 2 independent experiments (*n* = 10) for R-EAE. Since the effect for age was not significant, sham mice were analyzed as all from one population (*n* = 14 for mRNA and *n* = 8 for proteins). Data are shown as mean ± SEM. ****p < 0.0001; *p < 0.05

RT-qPCR was performed for two selected genes listed within the top ten upregulated genes in either R-EAE (Chi3l4) or TMEV-IDD (CXCL13) mice (Additional file 1: Table S1). For each gene, the increased expression initially suggested by microarray analysis was confirmed (Fig. 2a, b). CXCL13 mRNA levels were significantly higher in TMEV-IDD mice compared to R-EAE and sham mice (*p* < 0.0001) (Fig. 2a). Conversely, Chi3l4 gene expression was higher in R-EAE mice compared to both TMEV-IDD and sham mice (*p* = 0.031) (Fig. 2b).

CXCL13 and Chi3l4 ELISAs were then performed to quantitate protein levels, using spinal cord lysates as proteins’ source. As shown in Figure 2d and e, significant differences were observed in both CXCL13 and Chi3l4 levels between TMEV-IDD and R-EAE mice (both *p* ≤ 0.041) (Additional file 1: Table S1).

Production of IgG within the CNS was measured by using both a RT-qPCR assay for IgG1 mRNA and a Luminex assay for total IgG proteins in the spinal cord of TMEV-IDD and R-EAE mice (Additional file 1: Table S1). Levels of IgG1 mRNA in the spinal cord were significantly higher in TMEV-IDD mice compared with R-EAE mice (*p* < 0.0001) (Fig. 2c). In accordance, higher levels of total IgG were observed in the spinal cord of TMEV-IDD mice relative to R-EAE mice, although the difference was not significant (*p* = NS) (Fig. 2f).

### Phenotype and localization of B cells in the spinal cord of TMEV-IDD vs. R-EAE mice

**Fig. 3:**
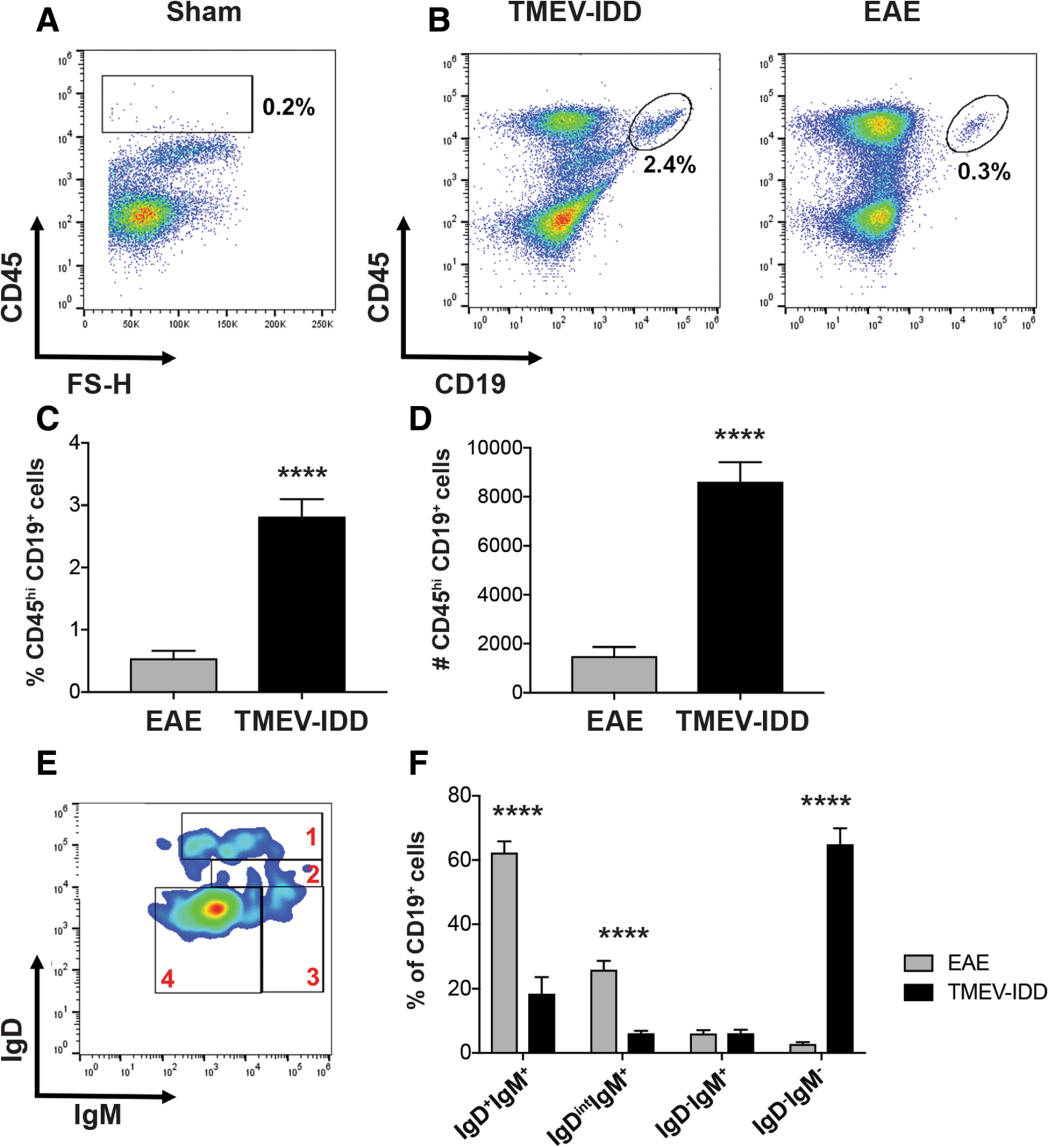
B cell accumulation in spinal cords of R-EAE and TMEV-IDD. Total B cells and B cell phenotypes defined by IgD and IgM surface marker expression were identified by flow cytometry in spinal cords from TMEV-IDD mice (*n* = 22) at day 120 post-infection or R-EAE mice (*n* = 12) at day 14 post-immunization. **a** Representative plot showing minimal CD45^hi^ infiltrating immune cells among total live cells in sham mice. **b** Representative gating strategy of CD45^hi^ CD19^+^ B cells among total live cells isolated from TMEV-IDD or R-EAE spinal cords. Bar graphs depict mean percentage ± SEM (**c**) and mean number ± SEM (**d**) of CD45^hi^ CD19^+^ B cells in TMEV-IDD or R-EAE spinal cords. **e** Representative smoothed plot discriminating IgD and IgM subsets including 1, IgD^+^IgM^+^ naïve/early activated; 2, IgD^int^IgM^+^ activated; 3, IgD^-^IgM^+^ transitional/ unswitched memory or ASC; and 4, IgD^-^IgM^-^ switched memory/ASC B cells within CD45^hi^ CD19^+^ cells in TMEV-IDD. **f** Bar graph depicting mean percentages ± SEM of B cell phenotypes discerned by IgD and IgM surface marker expression in TMEV-IDD and R-EAE. Statistical differences between TMEV-IDD and R-EAE spinal cords are indicated as determined by an unpaired *t* test. Data are representative of 3 independent experiments with 6 to 8 mice per experiments for TMEV-IDD and 2 independent experiments with 6 mice per experiment for R-EAE. ****p < 0.0001

Upregulated Ig- and B cell-related genes in TMEV-IDD suggested predominant B cell presence in CNS tissue during TMEV-IDD compared to R-EAE. Thus, we next sought to analyze alterations in B cell populations in the spinal cords of TMEV-IDD and R-EAE mice utilizing flow cytometry (Additional file 1: Table S1). Analysis of CD45^hi^ infiltrating immune cells revealed a marked increase in infiltrating cells in both models compared to sham mice (Fig. 3a, b), including accumulation of CD45^hi^CD19^+^ B cells in both TMEV-IDD and R-EAE spinal cords. However, quantification of B cell percentages and absolute numbers in TMEV-IDD and R-EAE tissues revealed three- to four-fold higher B cell infiltration in TMEV-IDD spinal cords (*p* < 0.0001) (Fig. 3c, d).

To characterize B cell phenotype, we then utilized IgD and IgM surface expression, identifying (1) IgD^+^IgM^+^ naïve/early activated B cells, (2) IgD^int^IgM^+^ activated B cells, (3) IgD^-^IgM^+^ transitional or isotype-unswitched antibody-secreting cells (ASC)/memory B cell (Bmem), and (4) IgD^-^IgM^-^ isotype-switched ASC/Bmem (Fig. 3e). Phenotypic comparison of spinal cord-infiltrating B cells in TMEV-IDD and R-EAE revealed significant differences in infiltrating B cell differentiation phenotype, with more than 80% of B cells in R-EAE spinal cords comprising a naïve/early activated or activated phenotype (*p* < 0.0001) (Fig. 3f). In contrast, more than 60% of spinal cord-infiltrating B cells in TMEV-IDD were identified as an isotype-switched ASC/Bmem phenotype (*p* < 0.0001).

**Fig. 4:**
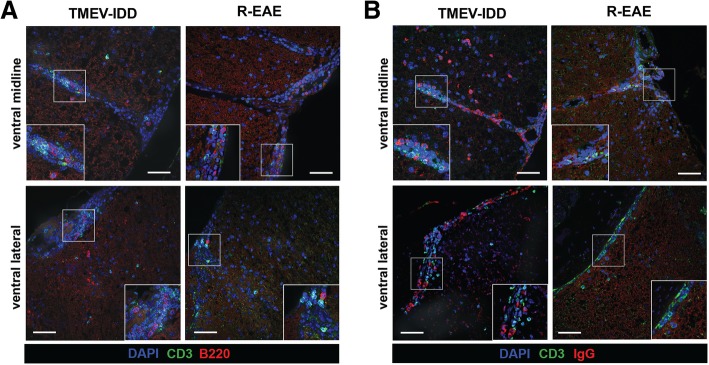
B cell localization in TMEV-IDD and R-EAE spinal cords. TMEV-IDD (day 120 post-infection) or R-EAE (day 14 post-immunization) decalcified spinal cords were stained with either **a** DAPI (blue), CD3 (green), and B220 (red) or **b** DAPI (blue), CD3 (green), and IgG (red) as indicated. Representative z stack projected compilations reveal immune cell aggregation in the ventral midline and ventral lateral meninges in TMEV-IDD and R-EAE mice, with minimal IgG immunostaining in R-EAE spinal cords. Insets in images show white boxed areas at higher magnification. Images are representative of 6 to 8 mice per group. Scale bar = 50 μm

We next sought to assess B cell localization in the CNS and aggregation with T cells, by analyzing B220 and CD3 expression (Additional file 1: Table S1). Immunostaining revealed infiltrating B220^+^ B cells and T cells aggregated to similar regions in TMEV-IDD and R-EAE, accumulating in the ventral midline and ventral lateral aspects of the spinal cord (Fig. 4a). However, flow cytometry also identified predominate infiltration of isotype-switched B cells in TMEV-IDD, including ASC which may downregulate B220 surface expression and thus become undetectable by B220 immunostaining. Thus, spinal cord sections were stained for the B cell marker IgG to assess localization of isotype-switched B cells and confirm minimal isotype-switched B cell accumulation in R-EAE. TMEV-IDD spinal cords revealed widespread parenchymal IgG^+^ B cell infiltration, with aggregation present in the ventral midline and ventral lateral meningeal regions of the spinal cord, often accumulating with CD3^+^ T cells. In contrast, minimal IgG^+^ immunoreactivity was observed in R-EAE spinal cords, with diffuse immunostaining proximal to the ventral midline, likely indicative of IgG leakage as a result of blood-brain barrier (BBB) permeability.

### B cell-related chemokines in serum and CSF of R-EAE and TMEV-IDD mice

**Fig. 5:**
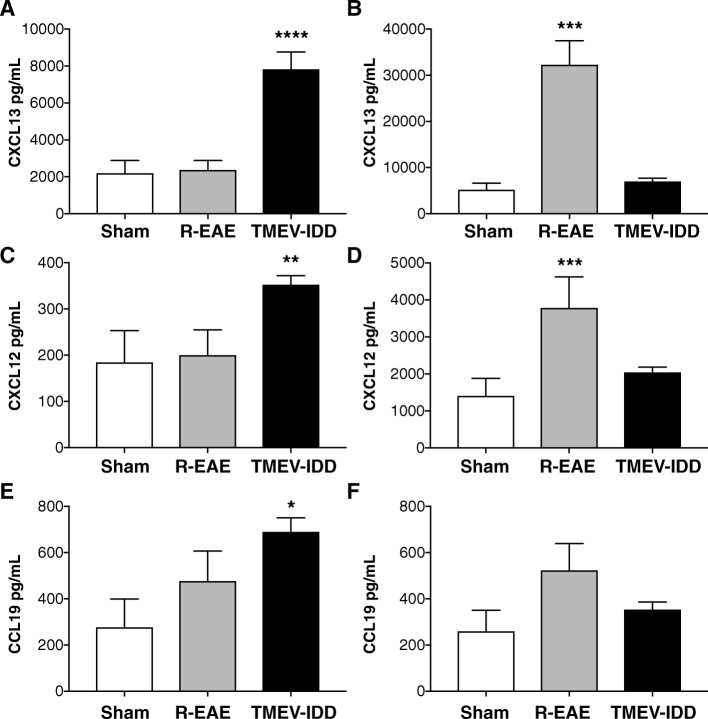
Chemokine levels (pg/mL) in CSF and serum of TMEV-IDD mice, R-EAE mice, and sham controls. The levels of CXCL13, CXCL12, and CCL19 were measured in individual CSF (**a**, **c**, **e**) and serum (**b**, **d**, **f**) specimens by Luminex bead immunoassays. CSF chemokine levels were higher in TMEV-IDD mice as compared to both R-EAE mice and sham controls. Conversely, serum chemokine levels were higher in R-EAE as compared to both TMEV-IDD mice and sham controls. Data are representative of 3 independent experiments (*n* = 11) for TMEV-IDD and 1 experiment (*n* = 6) for R-EAE. Since the effect for age was not significant, sham mice were analyzed as all from one population (*n* = 5). Data are shown as mean ± SEM. ****p < 0.0001; ***p < 0.001; **p < 0.01; *p < 0.05

Because microarray analysis, flow cytometry, and immunohistochemistry showed prominent involvement of B cells in TMEV-IDD, and to further understand mechanisms that influence B cell trafficking and accumulation in the CNS, we studied CSF and serum levels of some chemokines involved in B cell trafficking including CCL19, CXCL12, and CXCL13. CSF CCL19, CXCL12, and CXCL13 were all significantly elevated in TMEV-IDD mice as compared to both R-EAE mice and sham controls (all *p* ≤ 0.029) (Fig. 5a, c, e) (Additional file 1: Table S1).

Conversely, serum CXCL12 and CXCL13 were increased in R-EAE mice as compared to both TMEV-IDD and sham controls (both *p* ≤ 0.0004) (Fig. 5b, d). R-EAE mice also had higher serum CCL19 levels, but the difference was not statistically significant (*p* = 0.16) (Fig. 5f).

### Transudation and intrathecal synthesis of Igs in R-EAE and TMEV-IDD

**Fig. 6:**
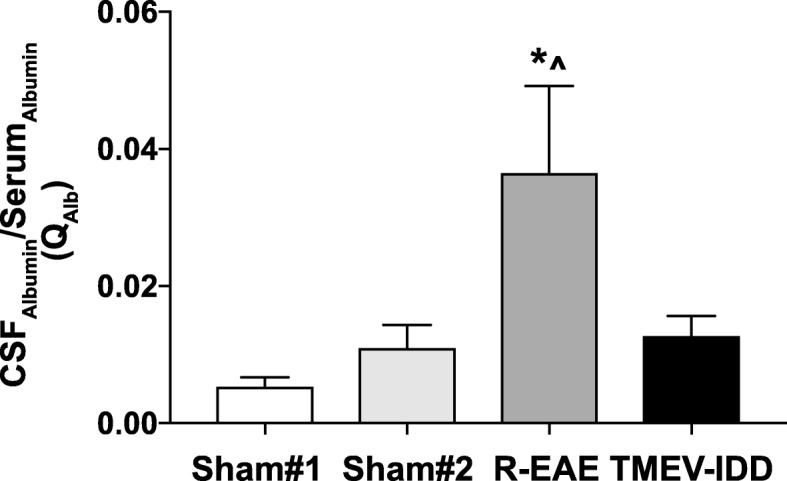
Blood-brain barrier function in R-EAE and TMEV-IDD. Albumin CSF/serum ratio evaluation (Q_Alb_) showed significantly increased values in R-EAE, which may reflect blood-brain barrier (BBB) breakdown in R-EAE mice. The integrity of the BBB permeability in TMEV-IDD is confirmed by the Q_albumin_ values similar to sham mice. Data are representative of 2 independent experiments (*n* = 26) for TMEV-IDD and 2 experiments (*n* = 22) for R-EAE. Since age may affect albumin expression and overall BBB permeability, 10-week-old sham SJL/J mice (sham#1, *n* = 4) and 24-week-old sham-treated SJL/J mice (sham#2, *n* = 12) were used as controls for R-EAE and TMEV-IDD, respectively. Values are expressed as mean ± SEM. *For comparisons between the disease model (either R-EAE or TMEV-IDD) and its age-matched sham control group. ^For comparisons between R-EAE and TMEV-IDD. *^*p* < 0.05

Multiplex analysis of different Ig subclasses and isotypes in the serum and CSF of individual mice was conducted to further confirm intrathecal antibody production in TMEV-IDD. Significant elevation of all the tested Igs was observed in the CSF of both R-EAE and TMEV-IDD mice when compared to sham controls (all *p* ≤ 0.038) (Additional file 1: Table S1). The CSF/serum albumin ratio (Q_albumin_) was then calculated to assess BBB function. We found a significant difference in Q_albumin_ (*p* = 0.0132); R-EAE mice showed higher values compared to both TMEV-IDD and sham mice (both *p* ≤ 0.021), indicating increased permeability of the BBB in R-EAE, but not in TMEV-IDD (Fig. 6). Additionally, there was no difference in Q_albumin_ between TMEV-infected and sham mice (*p* = 0.376), therefore confirming BBB integrity in TMEV-IDD. Similar conclusions resulted from a previous study from our group [20], which showed a lack of any measurable disruption of the BBB for either large or small molecules, as tested with both Q_albumin_ and Evans Blue, in chronic TMEV-IDD.

**Fig. 7:**
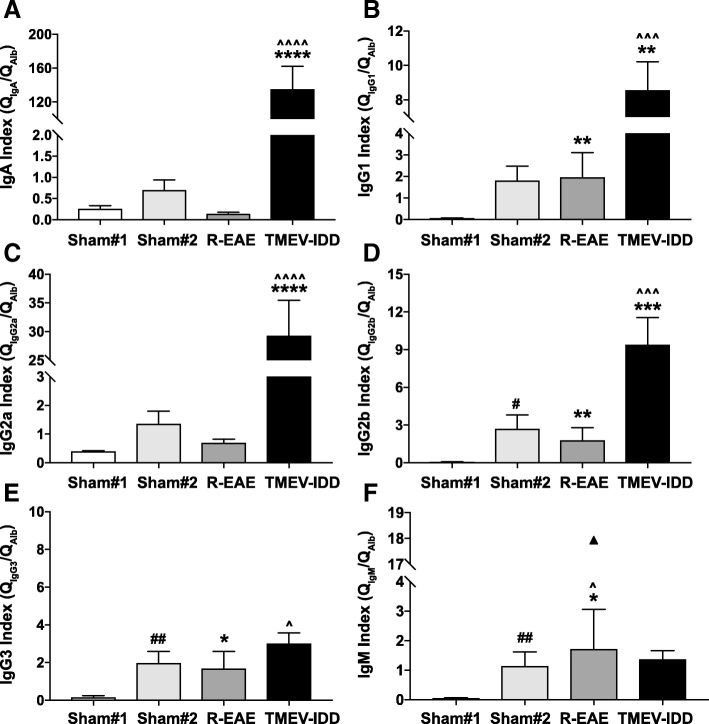
Intrathecal synthesis of immunoglobulins in R-EAE and TMEV-IDD. Indices (Q_Ig_/Q_albumin_) of all tested Igs (**a**–**d**), except for IgG3 and IgM (**e**, **f**), were significantly increased in TMEV-IDD mice compared to both R-EAE mice and age-matched sham controls (sham#2), reflecting significant intrathecal production of Igs. Data are representative of 2 independent experiments (*n* = 26) for TMEV-IDD and 2 experiments (*n* = 13) for R-EAE. Since the age of SJL/J mice could account for Ig levels, 10-week-old sham SJL/J mice (sham#1, *n* = 4) and 24-week-old sham-treated SJL/J mice (sham#2, *n* = 12) were used as controls for R-EAE and TMEV-IDD, respectively. Values are expressed as mean ± SEM. In **f**, an outlier is shown. *For comparisons between the disease model (either R-EAE or TMEV-IDD) and its age-matched sham control group. ^For comparisons between R-EAE and TMEV-IDD. #For comparisons between the two sham control groups. ****p < 0.0001; ***p < 0.001; **p < 0.01; *p < 0.05

To further discriminate between the passive transfer from the serum and intrathecally produced Igs, we also measured the ratio of Igs in CSF to serum (divided by the Q_albumin_ to correct for variance in BBB permeability) as an index of the endogenous production of Igs in the CNS. Indices of all Igs, except for IgG3 and IgM, were significantly increased in TMEV-IDD mice compared to both R-EAE and sham mice (all *p* ≤ 0.0047) indicating significant intrathecal Ig production in TMEV-IDD but not in R-EAE (Fig. 7).

## Discussion

In the above studies, we have compared two different murine models of MS. TMEV-IDD and R-EAE in the SJL mice are both relevant murine immune-mediated demyelinating models that recapitulate the two main MS disease phenotypes: TMEV-IDD serves as a virus-induced model of chronic progressive MS, whereas R-EAE closely mimics the clinical symptoms of relapsing-remitting MS (RRMS). This work has relevance to biological pathways differentially involved in these two forms of MS.

To determine the cellular and molecular changes associated with the differences in clinical course, a microarray analysis was carried out to identify genes with enriched expression in both models during the peak of their clinical disease, i.e., in the spinal cord of R-EAE mice during the peak of their first relapse as well as of TMEV-IDD mice during the late, chronic stage of their disease. Gene expression datasets from both models were compared to identify common and differential genes and pathways. A vast majority of genes mostly involved in innate/adaptive immunity pathways were similarly upregulated in the two models when compared to age-matched sham controls. However, we also identified three groups of genes that were regulated differently in R-EAE and TMEV-IDD.

The enrichment for genes associated with the regulation of axonogenesis, demyelination, and remyelination in the first gene cluster, i.e., sub-cluster #1, suggests active cycling between demyelination/remyelination and axonal regeneration in the spinal cord of R-EAE mice but not in the spinal cord of mice in the later stage of TMEV-IDD. The second gene cluster, i.e., sub-cluster #4, highlights, in R-EAE, a gene set enriched in processes related to adaptive immune responses, particularly T cell recruitment, activation, and differentiation. T cells are known to play a pivotal role in R-EAE as well as RRMS. Increased numbers of T cells are found in both the CNS and the periphery of R-EAE mice as well as in acute CNS lesions and CSF of MS patients during relapses [35, 36]. Therefore, our present data support the contribution of T cells in triggering clinical exacerbations.

The mechanism of CNS damage in MS has long been ascribed only to activated T cells that infiltrate the CNS and cause tissue injury [37]. Over the last decade, however, a significant amount of data has also implicated a dysregulated B cell biology in the pathogenesis of MS [38]. Although the pathogenesis of progressive MS remains unclear, B cells seem to play a significant role in chronic compartmentalized inflammation, likely contributing to disease progression [39]. Moreover, intrathecal Ig synthesis is associated with a worse long-term prognosis, greater disability, and faster MS disease progression [40–43]. Hence, most interesting was the enrichment in the third gene cluster, i.e., genes upregulated in TMEV-IDD as per sub-cluster #2, for genes involved in B cell activation and Ig synthesis. Since the increased expression of this specific gene cluster in TMEV-IDD may associate with dominant B cell presence in the CNS, we also compared alterations in B cell populations in the spinal cords of TMEV-IDD and R-EAE mice. Surprisingly, flow cytometric analysis revealed an increase in spinal cord-infiltrating B cells in both models, although to a lesser extent in R-EAE. This finding, which at first glance seems to contradict our gene expression data, implicates either a discrepancy between gene expression and protein translation or, more likely, phenotypic differences in the infiltrating B cells, underlying enhanced expression of genes involved in B cell activation and Ig production in TMEV-IDD compared to R-EAE. In line with the latter, a phenotypic comparison of the B cells infiltrating the spinal cords of the two models indicated an overall upregulation of markers typically associated with naïve/early activated B cells in R-EAE and with late differentiated B cells including ASC and Bmem in TMEV-IDD. These findings have important implications for functional differences in B cells infiltrating the CNS in R-EAE *vs*. TMEV-IDD. In R-EAE, a predominance of naïve/activated B cells suggests a possible role in antigen presentation [44–46] and/or pro- and anti-inflammatory cytokine production [47–49] in acute disease, as previously suggested in other acute EAE models. In contrast, during chronic TMEV-IDD, isotype-switched B cells, including Bmem and ASC phenotypes, preferentially accumulate in the CNS. Bmem and ASC, including plasmablasts and plasma cells, can produce pro- and anti-inflammatory cytokines [50–52] and engage in antigen presentation (only plasmablasts and Bmem) [53], while antibody production is unique to ASC. Interestingly, following stimulation, Bmem can rapidly convert into ASC to support ongoing antibody production [54, 55]. Our data thus show that alteration of the infiltrating B cell subsets and/or the Ig production capacities can be associated with the stage of the disease, i.e., acute and relapsing *vs*. chronic and progressive disease, eventually predicting disease evolution. B cells late in their differentiation sequence, i.e., plasma cells and Bmem cells, seem to preferentially accumulate in the CNS during a chronic progressive disease rather than during an acute inflammatory event. This concept is in line with previous findings showing that plasma cells and Bmem cells are commonly found in the chronically inflamed CNS [56], driving various chronic inflammatory conditions, including MS. Indeed, similar to what we describe in our experimental models, other authors have reported a trend toward an increase in the number of differentiated B cells in CNS lesions from patients with progressive MS in comparison to those with RRMS [9, 57].

Functionally, the B cell activation-related differences between R-EAE and TMEV-IDD may translate into an enhanced intrathecal antibody synthesis in progressive disease compared to acute relapses. While B cells aggregated to similar CNS regions in the two models, mostly accumulating in the ventral midline and ventral lateral aspects of the spinal cord, only TMEV-IDD exhibited high intrathecal Ig production. Several studies in patients seem to confirm this as an intrathecal synthesis of Igs, for example, is a crucial biological feature of MS, and a growing body of evidence suggests that it correlates with MS disease progression [40, 42, 43]. Moreover, a lack of correlation between CSF IgG levels and acute relapses in MS has been shown, while the intrathecal synthesis of Igs positively correlated with disease progression [41].

It remains unknown whether the B cells found in the CNS mature outside the CNS and then localize to the brain and spinal cord or whether the B cell differentiation occurs within the CNS. An intact BBB in TMEV-IDD mice along with the higher CSF levels of CXCL13, CXCL12, and CCL19 compared to matched serum samples provides evidence for an intrathecal synthesis of these chemokines mediating B cell localization to the CNS. Moreover, high Ig indices suggest antibody production is fostered within the CNS. Overall, these results indicate that the CNS may provide a niche for sustaining B cell responses independent of ongoing peripheral recruitment in progressive disease. In MS, B cell aggregation in the CNS is thought to perpetuate neuroinflammation through the local generation of pathogenic lymphocytes or autoantibodies [58]. B cell aggregates were found in the meninges of patients with progressive forms of MS and were associated with worse outcomes in patients [59, 60]. These aggregates exhibited some features reminiscent of B cell follicles in lymphoid tissue, including the presence of diverse B cell maturation phenotypes, B cell proliferation, and the presence of a stromal network producing B cell-related chemokines such as CXCL13 and CCL19 [60, 61]. Although we have yet to explore the contribution of B cell follicles in TMEV-IDD, our current studies support the notion that a compartmentalized B cell response occurs in the CNS of TMEV-IDD mice, but not in the CNS of R-EAE.

Conversely, in R-EAE, we observed a significant BBB breakdown, which is known to be a fundamental characteristic of EAE models [62], especially following PTx administration [63]. Concurrently, our results revealed that R-EAE mice had increased levels of CXCL13, CXCL12, and CCL19 in their serum, but not in their CSF. Elevations of these chemokines in the serum are consistent with an ongoing peripheral immune response, including the trafficking of B cells. Along the same line, the albumin quotient correlated with the Ig quotients suggesting that the CSF Ig elevation in these mice is mostly associated with a leaky BBB. These findings in R-EAE point toward ongoing peripheral B cell trafficking and activation that may have relevance to relapses. Similar to what has been previously described in PLP_139-151_-induced EAE studies in SJL mice [64], guinea pigs [65, 66], and rabbits [67], our data in R-EAE indicate that elevated CNS Ig levels, particularly oligoclonal IgG levels, are likely to be the result of a systemically primed humoral immune response toward a given antigen, without intrathecal Ig production. Although EAE is commonly considered a unique model for MS and as such has been largely used for studying overall MS disease pathogenesis, our findings reinforce the concept that PLP_139-151_-induced R-EAE in SJL mice mimics only partial aspects of MS, e.g, acute inflammation and relapses, which seem to be independent of intrathecal Ig synthesis, a key biological feature of MS. On the other hand, the intrathecal Ig synthesis observed in TMEV-IDD suggests that this model better mimics chronic inflammation in the CNS and thus progressive disease. Also, our findings in mice support the idea of a correlation between intrathecal Ig synthesis and disease progression in humans with MS. Finally, given that intrathecal Ig synthesis in TMEV-IDD results from the ongoing maintenance of B cells in the CNS compartment driven by a persistent viral infection of the CNS, and in line with previous evidence demonstrating an abnormal accumulation of Epstein-Barr virus (EBV)-infected B cells in the CNS of patients with progressive MS [27], our study provides support for the possible role for infectious agents in MS etiology.

## Conclusions

Overall, these findings, showing increased concentrations of intrathecally produced Igs as well as substantial infiltration of ASC in the CNS of TMEV-IDD mice, but not R-EAE mice, suggest a potential causative role for Igs and ASC in the chronic progressive phase of demyelinating diseases. Although further longitudinal studies in both TMEV-IDD and R-EAE will be necessary to reveal more complex disease patterns and their temporal characteristics, our present findings provide clear insight into the major pathological differences during relapsing and progressive phases of MS. These findings could have important clinical implications, given the current paucity of treatment options for people with progressive MS.

## Additional files

Additional file 1: **Table S1.** Clinical and experimental characteristics of mice. Mice information. (DOCX 24 kb)

Additional file 2: **Table S2.** Gene expression changes in R-EAE (E) and TMEV-IDD (T) versus their respective age-matched controls (Sham_E and Sham_T). Microarray data. (XLSX 2720 kb)

Additional file 3: **Table S3.** Subclusters of genes/probes showing the most differential expression patterns between TMEV-IDD and R-EAE. Microarray data. (XLSX 111 kb)

Additional file 4: **Table S4.** Gene ontology enrichment analysis in subcluster #1. Microarray data. (XLSX 29 kb)

## Abbreviations

ASC: Antibody secreting cells
BBB: Mlood-brain barrier
Bmem: Memory B cell
CCL9: Chemokine (C-C motif) ligand 19
CFA: Complete Freund’s adjuvant
Chi3l4: Chitinase-3-like protein 4
CNS: Central nervous system
CPE: Cytopathic effect assay
p.i: Post-infection
CSF: Cerebrospinal fluid
CXCL13/12: Chemokine (C-X-C motif) ligand 13/12
DAVID: Database for Annotation, Visualization, and Integrated Discovery
DT: Decision trees
FACS: Fluorescence-activated cell sorter
GAPDH: Glyceraldehyde phosphate dehydrogenase
GO: Gene ontology
IACUC: Institutional Animal Care and Use Committee
Ig: Immunoglobulin
LOOCV: Leave-one-out cross-validation
MS: Multiple Sclerosis
PBS: Phosphate-buffered saline
PFU: Plaque-forming units
PLP: Proteolipid protein peptide
PTx: Pertussis toxin
Q_albumin_: Albumin quotient
R-EAE: Relapsing experimental allergic encephalomyelitis
RRMS: Relapsing-remitting MS
RT-qPCR: Real-time reverse transcription quantitative PCR
SAM: Statistical analysis of microarrays
Th: T helper
TMEV-IDD: Theiler’s murine encephalomyelitis virus-induced demyelinating disease
