# Supplementary material for: Differential neuro-immune patterns in two clinically relevant murine models of multiple sclerosis
Source: J Neuroinflammation. 2019 May 22;16:109. doi: 10.1186/s12974-019-1501-9 (PMC6532235; doi:10.1186/s12974-019-1501-9)
Supplement: Supplementary file 1 — Table S1. Clinical and experimental characteristics of mice. Mice information. (DOCX 24 kb) [file 12974_2019_1501_MOESM1_ESM.docx]

**Additional file 1: Table S1: Clinical and Experimental Characteristics of mice.**

|  |  | **R-EAE** | **Sham #1** | **TMEV-IDD** | **Sham #2** |
| --- | --- | --- | --- | --- | --- |
| ***MicroArray Analysis*** | *n.* | 4 | 4 | 4 | 4 |
|  | *Age (Av. weeks)* | 9.5 | 9.5 | 27.4 | 27.4 |
|  | *Days p.i. (Av. Days)* | 12 | 12 | 150 | 150 |
|  | *Clinical Score -Mean (Range)* | 3.1 (2.0-4.0) | 0 | 0.33 (0.16-0.36) | 1.38 (0.77-1.85) |
| ***CXCL13, Chi3l4, IgG1 RT-qPCR*** | *n.* | 16 | 6 | 31 | 8 |
|  | *Age (Av. weeks)* | 9.8 | 10.4 | 23.4 | 23.8 |
|  | *Post-immunization (Av. Days)* | 13 | 17 | 122 | 125 |
|  | *Clinical Score -Mean (Range)* | 3.6 (2.5-4.0) | 0 | 0.47 (0.11-0.85) | 1.24 (1.13-1.56) |
| ***CxCL13, Chi3l4, IgG Protein Analysis*** | *n.* | 10 | 3 | 15 | 5 |
|  | *Age (Av. weeks)* | 10.4 | 10.4 | 23 | 23.1 |
|  | *Post-immunization (Av. Days)* | 17 | 17 | 119 | 120 |
|  | *Clinical Score -Mean (Range)* | 2.6 (1.5-3.5) | 0 | 0.41 (0.05-1.15) | 1.47 (1.36-1.61) |
| ***Flow Cytometry*** | *n.* | 12 | 6 | 22 | 6 |
|  | *Age (Av. weeks)* | 10 | 10.4 | 23.1 | 23.1 |
|  | *Post-immunization (Av. Days)* | 14 | 17 | 120 | 120 |
|  | *Clinical Score -Mean (Range)* | 3.05 (0.5-4.5) | 0 | NT | NT |
| ***Histology*** | *n.* | 8 | 6 | 8 | 6 |
|  | *Age (Av. weeks)* | 9.8 | 10.4 | 23.8 | 23.1 |
|  | *Post-immunization (Av. Days)* | 13 | 17 | 125 | 120 |
|  | *Clinical Score -Mean (Range)* | 3.6 (1.5-4.5) | 0 | 0.62 (0.31-0.71) | NT |
| ***Chemokine Luminex Analysis*** | *n.* | 12 | 6 | 22 | 6 |
|  | *Age (Av. weeks)* | 10 | 10.4 | 23.1 | 23.1 |
|  | *Post-immunization (Av. Days)* | 14 | 17 | 120 | 120 |
|  | *Clinical Score -Mean (Range)* | 3.05 (1.5-4.5) | 0 | NT | NT |
| ***BBB Permeability Analysis*** | *n.* | 22 | 4 | 26 | 12 |
|  | *Age (Av. weeks)* | 9.8 | 9.7 | 122 | 122 |
|  | *Post-immunization (Av. Days)* | 13 | 12 | 23.4 | 23.4 |
|  | *Clinical Score Mean (Range)* | 3.05 (1.5-4.0) | 0 | 0.51 (0.21-1.15) | 1.62 (1.46-1.85) |

Clinical signs in R-EAE were assessed on a six stage scale of 0–5; in TMEV-IDD mice, long-term monitoring for disability progression was accomplished with the Rotarod performance test, expressing the data as a neurological function index (NFI). Av.=average; NT=not tested. BBB= blood-brain barrier; Days p.i. stands for “days post-immunization” in R-EAE mice and “days post-infection” in TMEV-IDD mice.
